# Developing patient-refined colorectal cancer screening materials: application of a virtual community engagement approach

**DOI:** 10.1186/s12876-023-02774-8

**Published:** 2023-05-24

**Authors:** Priyanka Gautom, Anne L. Escaron, Joanna Garcia, Jamie H. Thompson, Jennifer S. Rivelli, Esmeralda Ruiz, Evelyn Torres-Ozadali, Dawn M. Richardson, Gloria D. Coronado

**Affiliations:** 1 OHSU-PSU School of Public Health, 1810 SW 5th Ave, Portland, OR 97201 USA; 2 Institute for Health Equity, AltaMed Health Services Corp, 2040 Camfield Avenue, Los Angeles, CA 90040 USA; 3 Kaiser Permanente Center for Health Research, 3800 N. Interstate Avenue, Portland, OR 97227 USA

**Keywords:** Community engagement, Virtual platform, Boot camp translation, Colorectal cancer screening, Federally qualified health center

## Abstract

**Introduction:**

In partnership with a federally qualified health center (FQHC), an adapted virtual version of boot camp translation (BCT) was used to elicit input from Spanish-speaking Latino patients and staff to develop messaging and patient education materials for follow-up colonoscopy after abnormal fecal testing. We describe how we adapted an existing in-person BCT process to be delivered virtually and present evaluations from participants on the virtual format.

**Methods:**

Three virtual BCT sessions were facilitated by bilingual staff and conducted via Zoom. These sessions included introductions and discussions on colorectal cancer (CRC), CRC screening, and gathered feedback from participants on draft materials. Ten adults were recruited from the FQHC. A research team member from the FQHC served as the point of contact (POC) for all participants and offered Zoom introductory sessions and/or technology support before and during the sessions. Following the third session, participants were invited to complete an evaluation form about their virtual BCT experience. Using a 5-point Likert Scale (where 5 = strongly agree), questions focused on session utility, group comfort level, session pacing, and overall sense of accomplishment.

**Results:**

Average scores ranged from 4.3 to 5.0 indicating strong support towards the virtual BCT sessions. Additionally, our study emphasized the importance of a POC to provide technical support to participants throughout the process. Using this approach, we successfully incorporated feedback from participants to design culturally relevant materials to promote follow-up colonoscopy.

**Conclusion:**

We recommend ongoing public health emphasis on the use of virtual platforms for community engaged work.

**Supplementary Information:**

The online version contains supplementary material available at 10.1186/s12876-023-02774-8.

## Introduction

In the United States, colorectal cancer (CRC) is one of the leading causes of cancer death and is estimated to account for more than  52,000 deaths in 2023 [1]. Current guidelines recommend that adults between the ages of 45–75 be routinely screened for CRC through one of the several different screening methods, including colonoscopy, flexible sigmoidoscopy, and CT colonography and stool tests such as the guaic-based fecal occult blood test (gFOBT), fecal immunochemical test (FIT), and FIT-DNA [2].

Early diagnosis of CRC from screening is vital as it increases survival from the disease [3]. Assessments report that 68.8% of American adults aged 50 to 75 were up-to-date with CRC screening in 2018, however, CRC related incidence and mortality vary by race and ethnicity which can be partially attributed differences in screening. CRC screening rates among Latino Americans is much lower when compared to White Americans, where 56.1% and 71.1%, respectively, of age eligible adults were up-to date [4, 5]. Furthermore, care disruptions during the COVID-19 pandemic led to further reductions in CRC screening participation. Estimates show an abrupt decline in CRC screening between March and June 2020, totaling to nearly 95,000 missed screenings [6]. This is quite concerning, as the backlog of screenings in addition to the new barriers to complete screening caused by COVID-19 may further exacerbate the CRC screening and mortality inequities impacting Latinos.

There are several barriers to increasing CRC screening within the Latino population. Studies have found that lack of CRC screening was associated with younger age groups (50–54 vs. 70–75), low patient awareness about CRC and the need for screening, absence of insurance, fear of screening results, limited access to health providers, and language barriers [7–11]. Effective messaging and materials are important in overcoming these challenges and can aid in communicating the importance of timely screening [12, 13].

Engaging patients and community members in research can help communicate cancer screening guidelines in a way that meets patients’ social, linguistic, and cultural needs [14]. The rise of telehealth and other virtual healthcare services as a response to disruption of regular in-person care creates a unique opportunity to translate community engagement processes to virtual settings. Shifting to online platforms can be an important strategy in the context of the current pandemic and beyond as it will ensure preventive services continue during the disruption of normal operations.

**Fig. 1:**
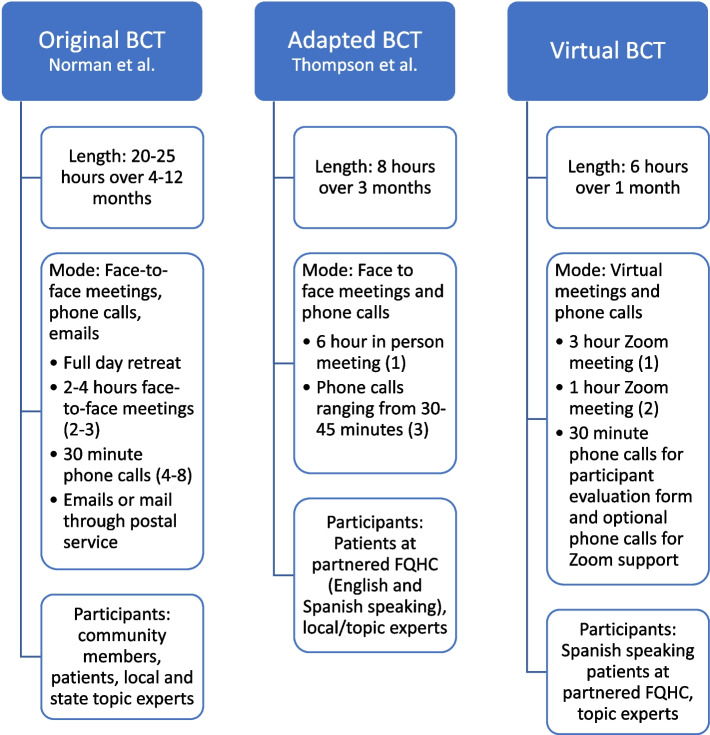
Summary of BCT processes

Boot camp translation (BCT) is a validated method of engaging patients to refine educational materials and can be used to translate evidence-based recommendations in ways that are understood by and important to communities [15]. Medical and scientific jargon can be a barrier to both understanding health recommendations and to conversing with healthcare providers regarding treatment options, making BCT an effective tool in increasing communication between patients and their health team [16]. BCT has previously been successful in adapting culturally relevant health messages promoting FIT testing for Latino patients through in-person workshops [17]. However, to the best of our knowledge, no prior report on BCT in a virtual setting has been published. We conducted a virtual BCT (due to COVID-19 restrictions) to elicit information from Spanish-speaking Latino patients and health center staff. Our goal was to create culturally tailored messages and patient education materials to promote follow-up colonoscopy after abnormal fecal testing. Here we describe how we adapted an existing in-person BCT process to be delivered virtually. We also present brief evaluations from patients and health center staff on the virtual format. Our findings can inform future virtual patient engagement activities for low-literacy and non-English speaking populations. Figure 1 provides a summary of the original BCT processes, the adapted in-person process previously used by our research team, and the virtual process reported here.

## Methods

### Participant recruitment

In this study, we partnered with a large independent federally qualified health center (FQHC) in Los Angeles and Orange Counties to engage Latino patients and health center staff in a virtual adaption of BCT. Our virtual BCT sought to create messages to increase follow-up colonoscopy screenings after an abnormal fecal immunochemical test (FIT) result within the Latino community. The sessions were conducted in Spanish because it was the primary language spoken by the FQHC’s age-eligible patient population. Zoom for Healthcare was chosen as the platform for the virtual sessions based on its usability and HIPAA compliancy. Virtual sessions were not recorded, in keeping with patient privacy recommendations.

Due to existing patient relationships, the FQHC was responsible for recruiting study participants. Participants were identified in the electronic health record with the following criteria: identified as Latino, Spanish speaking, between the ages of 50 and 75, without a personal history of CRC or colorectal disease, completion of a FIT, and the ability to participate in the three online sessions. Language was pulled from the electronic health record and clinic study staff also asked participants for their preferred language. Patients were eligible irrespective of their FIT result and/or whether they obtained a follow-up colonoscopy.

Using Microsoft Word’s feature for language assessment, recruitment materials were written at a 5^th^ grade reading level with the priority population in mind and were written in both English and Spanish. Recruitment flyers explaining the study were mailed to the homes of patients who met the eligibility requirements. An initial batch of 60 recruitment flyers were mailed followed one week later by a phone call from a member of the FQHC’s research team, acting as the main point of contact (POC) for patient correspondence. Two weeks after the initial mailing, FQHC staff mailed a second batch of recruitment flyers (*n* = 60) and followed up with a phone call due to low enrollment. FQHC staff had trouble reaching patients via telephone and four patients were enrolled after phone calls placed to the first batch of recruitment flyer recipients. The POC conducted calls during normal business hours (8 am to 5 pm) and on two Saturday mornings (9 am to 11 am) to accommodate prospective participants’ work schedules. If patients expressed interest during the phone call, the POC confirmed study eligibility and enrolled the patient into the study. The POC confirmed the participant’s mailing address, their preferred mode of communication, preferred language for health communication, and assessed their needs for accessibility to online sessions.

**Fig. 2:**
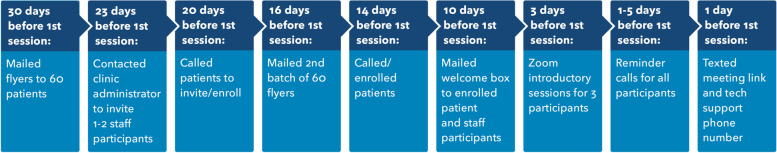
Summary of Recruitment Activities

Next, a welcome box was mailed to all enrolled participants with presentation materials, snacks, a stress toy, notebooks, pens, and a recruitment flyer that included information about study logistics. A week after the box was mailed, the POC called each of the participants to confirm they had received the box and to review the contents. Additionally, the week prior to the first virtual session, the POC conducted one-on-one meetings with patient participants who were unfamiliar with digital conference platforms, such as Zoom, or without a family member to assist them. In preparation for this connectivity session, the POC texted a link to patients including Zoom connectivity how-to materials. One day before the session, a final reminder was sent via text message with the link and instructions to join the Zoom meeting. The text also included a phone number for a different FQHC staff person to call if participants encountered any difficulties while trying to log on to Zoom the day of the session. Staff participants were sent the session link via their calendar routinely used for work functions. The timeline of recruitment activities conducted by the research team’s POC is summarized below in Fig. 2. Participants were given up to $150 in gift cards for participating ($100 for the three-hour session, $25 for the first follow-up session and $25 for the second follow-up session). The three BCT sessions took place between May and June 2021. While the main study is registered on ClinicalTrials.gov (NCT03167125), the BCT process implemented for this work has minimal risk. Due to the minimal risk of the BCT process, the Kaiser Permanente Northwest IRB waived the requirement for informed consent and the need for privacy rule authorization to access protected health information.

The initial 3-hour session was facilitated by a member of the research team who is a bilingual (English and Spanish) and bicultural mental health counselor, and has been trained in BCT facilitation by the High Plains Research Network. We developed case studies that showcased typical reported patient barriers to follow up screening to frame the discussions in a relatable way. All sessions were conducted in Spanish by the aforementioned bilingual/bicultural trained professional. Bilingual status was assessed through the researcher’s organization’s training program which includes a language certification process. This approach allowed us to understand cultural concepts, the meaning of phrases and idioms, and provided linguistic concordance. This linguistic and cultural tailoring fostered trust and sharing of colorectal cancer screening experiences throughout the sessions as well as maintaining participant engagement as participants reported enjoying the group discussions. Additionally, this allows bilingual/bicultural staff to complement the research process and create greater depth within the research [18]. 

### Session set-up

Prior to the participant sessions, a mock-session with the multi-site research team was conducted to review the slide deck, confirm that the audio from embedded videos could be heard, and confirm the breakout room assignment process. The sessions were organized as follows:

1. Session #1 (three-hours) took place on a weekend per participant preference and consisted of two expert presentations delivered in Spanish by the bilingual study PI. These presentations included animated videos explaining colon health, colon cancer, screening pathways, and steps to getting a colonoscopy. In addition, barriers to colonoscopy and effective messages were discussed. To increase interaction, participants were encouraged to ask questions and give comments throughout the session. Additionally, breakout rooms were used to create smaller group settings to elicit more discussion regarding colonoscopy messaging (see Additional file 1: Appendix for the agenda of session #1). This main session included five roles which were assigned to the bilingual (English and Spanish) members from the research team. The roles included: one expert presenter, one facilitator, one designated tech support person, note takers, and two attendance keepers. Each breakout room included a facilitator and notetaker. Additionally, a waiting room within the Zoom platform was used to confirm the identity of participants.
2. Sessions #2 and #3 (one-hour each) showcased colonoscopy related videos and draft fact sheets to garner feedback and suggestions from participants to finalize follow-up colonoscopy messaging materials. These sessions included one facilitator, one notetaker, one designated tech support person, and one attendance keeper.

### Data collection and analysis

Following the third session, participants were invited by phone to complete an evaluation form about their virtual BCT experience. Questions were presented using a 1 to 5-point Likert Scale (5 = strongly agree) that focused on session utility, group comfort level (felt listened to and felt comfortable sharing new ideas), session pacing, and overall sense of accomplishment. Two open-ended questions queried about the most helpful part of the session and ways to improve the session.

## Results

### Participant recruitment

A total of 120 study enrollment flyers were mailed to eligible participants and 90 successful follow-up calls were made, where the POC spoke directly with people or left voicemails to active phone numbers. Fifteen total participants, which included twelve community members and three staff members from the FQHC, were enrolled in the study. The three clinic staff participants were recruited after the project team emailed Clinic Administrators to invite staff to participate in the sessions. All participants identified as women between the ages of 57 and 67 and indicated their preferred language is Spanish. Of the total 15 enrolled, ten attended Session #1 (seven community members and three clinic staff). One patient misunderstood the instructions to participate in the virtual session and showed up in person at an Orange County clinic. The most common reason why interested eligible patients were unable to join the virtual sessions was due to session timing as several people stated they worked weekends and were unable to take the time off to participate. They also expressed that the COVID-19 pandemic had detrimentally affected their jobs and they could not afford to take a day off to join the session since job security was at stake. One patient participant started a job after the first session and was unable to return for the two remaining sessions. Seven participants attended Session #2 (four community members and three clinic staff) while eight attended Session #3 (five community members and three clinic staff).

### Virtual session engagement

Three patient participants requested a separate appointment with the POC prior to the first session for an introduction to Zoom. During this meeting, the POC guided participants on the steps to download the Zoom application to their device, gave instructions on how to connect to the session, and explained how to join the session using the camera and microphone features. The POC also communicated with different family members, usually English-speaking adult children and English-speaking school-aged grandchildren, who were familiar with Zoom and able to set up the meeting on the day of the session. A FQHC team member was appointed as technical support for each of the sessions and their phone number was sent to the participants the day before the initial session; however, participants needing technical help contacted the POC directly instead of the designated technical support person.

One of the participants did not have access to a smartphone or computer/tablet and instead used the Zoom audio call-in function. This participant was able to follow along with the presentation by referencing the printed materials included in the welcome box. Even after the introductory Zoom meeting, two participants experienced significant technology difficulties with connecting to all three sessions. Each time, the participants were able to successfully join the sessions after troubleshooting with the POC. Most of the patient participants used the camera feature throughout the session; four of the seven participants had their cameras on at all times. All seven participants utilized the mute/unmute feature on Zoom to speak during the session.

### Participant experience

Evaluation form summary scores indicated strong support of and positivity towards the virtual BCT session. Average scores ranged from 4.3 to 5.0 for six patients and three staff. Two staff reported audio issues, which impacted their comfort level score, as they had trouble being heard and sharing new ideas. In response to the open-ended question about which part of the session participants found to be most helpful, three of six patients said they found all parts of the session most helpful; two other patients identified the videos as being most helpful; another reported learning about early stages of detecting colon cancer; a patient also suggested that the follow up colonoscopy exam should be discussed during the clinical encounter. In contrast, for the staff attendees, hearing patients’ point of view and perspective was most helpful. In terms of improving the sessions, two of six patients raised low attendance; two suggested an in-person meeting; and two had no suggestions. One patient who had started a new job cited scheduling as an area for improvement and another patient had interest in sessions on other topics including nutrition and diabetes. Staff each raised different areas for improvement: staff suggested a future in-person meeting (as tech issues were encountered during the virtual session); a longer second session; and having male participants in the sessions. Overall, BCT participants demonstrated strong levels of engagement throughout the virtual sessions and were satisfied with the virtual platform.

## Discussion

The goal of this study was to engage Spanish-speaking Latino patients and health center staff to develop messaging and patient education materials to encourage follow-up colonoscopy after abnormal fecal testing. We adapted an existing patient engagement process, BCT, to a virtual format to refine Spanish-language materials with Latino patients at a large FQHC headquartered in Los Angeles. To continue community engaged work during a time when in-person meetings were disrupted, the adapted BCT held three virtual sessions on Zoom with patients and clinic staff. The adapted virtual BCT described here, although small in scale, may be a vital strategy contributing to community engaged research throughout the post-pandemic period.

The research team’s POC offered one-on-one Zoom introductory sessions to all participants and met with three of the participants prior to the first meeting. Additionally, the POC spoke to family members to assist with any technology troubleshooting during the sessions. During the sessions, three participants encountered technical difficulties, which were resolved after contacting the POC.

We administered a post-call evaluation form to attendees to inform future efforts, and responses indicated strong satisfaction with the virtual sessions. Overall, participants felt comfortable in the sessions, but there were some technological difficulties. Ideas for improvement included involving more patients in the sessions, having male participants, and in-person meetings to minimize technology issues.

### Lessons learned

Compared to in-person approaches, we learned that limiting the group size and shortening the number of hours of the BCT sessions were necessary to accommodate the Zoom platform. This was to ensure participants were engaged throughout the sessions. Also, we were unable to recruit male participants which may limit the response to the materials and messages we developed. In future video-conference-based BCT sessions, we might consider holding multiple smaller sessions that focus on subgroups of interest (e.g., all women and all men). This way, we can assure that our messages and materials have a broad appeal.

Our study contributes to the growing research on strategies to conduct patient engagement activities using virtual platforms. During the pandemic, various programs found that parts of community engaged work can successfully happen through virtual approaches. Shifting to virtual approaches allowed for an increase in the ability to reach community participants geographically and increased flexibility by eliminating barriers to attendance like transportation and childcare while sustaining the same quality of data as in-person engagements. However, many of these programs found that community members prefer in-person engagement to build connections [19, 20]. Finding different modes of participatory research is imperative to ensure continuation of patient engagement.

### Strengths and limitations

A key strength of our study was the use of bilingual study staff which allowed the team to establish a common cultural understanding with the participants to ensure their experiences and health needs were heard and acted on. Moreover, clinic staff who recruited participants were able to adjust their strategy to reach our recruitment targets.

While moving to a virtual setting did alleviate barriers caused by the inability to meet in person due to the pandemic, there have been documented challenges in engaging older Latino adults virtually, as they may have limitations to online communications [21]. One of the main strengths of our study was the ability to reach our sample of Spanish-speaking participants. The participants were engaged throughout the sessions, provided input on their learnings and suggestions for materials, and overall enjoyed the virtual BCT process. However, several potential participants were unable to participate due to lack of access to a computer or Zoom, thus, the generalizability of our findings may be limited to individuals who are either comfortable with a computer/smart phone or have household members who are able to help them. Additionally, the relatively high scores given to the quantitative participant experience questions may include a level of response bias as the patients and staff of the FQHC were known and may have wanted to provide socially desirable responses.

The success of virtual BCT is limited by the reach of participants who can attend, and while we were able to reach our recruitment target, the information gathered from the sessions may not extend beyond the specific population included in this study. Furthermore, the study included only women from an urban setting, making it difficult to generalize the findings to the larger Latino population. We recommend a more representative study, including participants across genders, to better understand factors related to the reach of virtual engaged work. Overall, our study demonstrates that it is feasible to offer adapted BCT in a virtual format and emphasizes the importance of a technical point of contact (POC) to provide support to potential participants.

## Conclusion

Despite the challenges of adapting to a virtual format and the possibility of selection bias, community engagement continues to be important to public health. Our findings show that Spanish-speaking Latino patients and health center staff were able to engage in a virtual session and they had favorable reactions to the virtual program. We recommend ongoing public health emphasis on the use of virtual platforms for community engaged work in times when in-person engagements are not available.

## Supplementary Information

**Additional file 1: Appendix.** Virtual BCT Session #1 Agenda.

## Data Availability

The materials developed by the current study are available at www.mailedFIT.org.

## Abbreviations

FQHC: Federally qualified health center
BCT: Boot camp translation
CRC: Colorectal cancer
POC: Point of contact
FIT: Fecal immunochemical test
gFOBT: Guaic-based fecal occult blood test
