# Supplementary material for: Developing patient-refined colorectal cancer screening materials: application of a virtual community engagement approach
Source: BMC Gastroenterol. 2023 May 24;23:179. doi: 10.1186/s12876-023-02774-8 (PMC10205160; doi:10.1186/s12876-023-02774-8)
Supplement: Supplementary file 1 — Additional file 1: Appendix. Virtual BCT Session #1 Agenda. [file 12876_2023_2774_MOESM1_ESM.docx]

**Appendix**

**Virtual BCT Session #1 Agenda**

9:30 am – 10:00 am Welcome + Introductions

10:00 am – 10:45 am Expert Presentation #1

- - - - - *Colorectal cancer and screening*
        - *Getting a colonoscopy*

10:45 am – 11:00 am Group Discussion

11:00 am – 11:10 am Break

11:10 am – 11:30 am Expert Presentation #2

- *Motivating patients to get a colonoscopy*

11:30 am – 12:15 pm Small Group Sessions (2 breakout groups)

12:15 pm – 12:30 pm Wrap-up
